# The synthetic killer peptide KP impairs *Candida albicans* biofilm *in vitro*

**DOI:** 10.1371/journal.pone.0181278

**Published:** 2017-07-13

**Authors:** Simona Paulone, Andrea Ardizzoni, Arianna Tavanti, Serena Piccinelli, Cosmeri Rizzato, Antonella Lupetti, Bruna Colombari, Eva Pericolini, Luciano Polonelli, Walter Magliani, Stefania Conti, Brunella Posteraro, Claudio Cermelli, Elisabetta Blasi, Samuele Peppoloni

**Affiliations:** 1 Department of Diagnostics, Clinical and Public Health Medicine, University of Modena and Reggio Emilia, Modena, Italy; 2 Department of Biology, University of Pisa, Pisa, Italy; 3 Department of Translational Research and New Technologies in Medicine and Surgery, University of Pisa, Pisa, Italy; 4 Department of Medicine and Surgery, University of Parma, Parma, Italy; 5 Institute of Public Health, Università Cattolica del Sacro Cuore, Fondazione Policlinico Universitario Agostino Gemelli, Rome, Italy; Venenum Biodesign, UNITED STATES

## Abstract

*Candida albicans* is a commensal organism, commonly inhabiting mucosal surfaces of healthy individuals, as a part of the resident microbiota. However, in susceptible hosts, especially hospitalized and/or immunocompromised patients, it may cause a wide range of infections. The presence of abiotic substrates, such as central venous or urinary catheters, provides an additional niche for *Candida* attachment and persistence, particularly via biofilm development. Furthermore, *Candida* biofilm is poorly susceptible to most antifungals, including azoles. Here we investigated the effects of a synthetic killer peptide (KP), known to be active *in vitro*, *ex vivo* and/or *in vivo* against different pathogens, on *C*. *albicans* biofilm. Together with a scrambled peptide used as a negative control, KP was tested against *Candida* biofilm at different stages of development. A reference strain, two fluconazole-resistant and two fluconazole-susceptible *C*. *albicans* clinical isolates were used. KP-induced *C*. *albicans* oxidative stress response and membrane permeability were also analysed. Moreover, the effect of KP on transcriptional profiles of *C*. *albicans* genes involved in different stages of biofilm development, such as cell adhesion, hyphal development and extracellular matrix production, was evaluated. Our results clearly show that the treatment with KP strongly affected the capacity of *C*. *albicans* to form biofilm and significantly impairs preformed mature biofilm. KP treatment resulted in an increase in *C*. *albicans* oxidative stress response and membrane permeability; also, biofilm-related genes expression was significantly reduced. Comparable inhibitory effects were observed in all the strains employed, irrespective of their resistance or susceptibility to fluconazole. Finally, KP-mediated inhibitory effects were observed also against a catheter-associated *C*. *albicans* biofilm. This study provides the first evidence on the KP effectiveness against *C*. *albicans* biofilm, suggesting that KP may be considered as a potential novel tool for treatment and prevention of biofilm-related *C*. *albicans* infections.

## Introduction

*Candida albicans* is a commensal microorganism, commonly found in healthy individuals as a member of skin, gastrointestinal or vaginal microbiota. Nevertheless, it becomes one of the major fungal pathogens in critically ill patients and immunocompromised individuals, where it causes severe, often life-threatening, deep-seated infections [1, 2]. This yeast has the capability to organize into structured microbial communities, known as biofilm, on abiotic (i.e. catheters and other medical devices) or biotic (i.e. oral mucosae) surfaces [3–5]. In particular, *C*. *albicans* produces a three-dimensional community composed of multiple cell types (round budding yeast cells, oval pseudo-hyphal cells and elongated hyphal cells) embedded in a matrix of extracellular polysaccharides [6]. Importantly, once structured as biofilm, *C*. *albicans* exhibits enhanced tolerance to antifungal therapy and host defence mechanisms as well. Consequently, medical device-associated biofilms are often accompanied by failure of conventional therapy; even more, they behave as a reservoir for persistent infections [7–9]. Because of fungal biofilm resilience to antifungals, therapeutic approaches are often limited. Therefore, it is necessary to identify new effective compounds/strategies against *C*. *albicans* biofilm. Antimicrobial peptides have recently been investigated as novel and potentially effective anti-biofilm compounds[10, 11].

Killer peptide (KP) is a well-known decapeptide derived from the sequence of the variable region of a single-chain recombinant anti-idiotypic antibody that represents the functional internal image of a wide-spectrum fungicidal yeast killer toxin targeting β-1,3-ᴅ-glucan cell-wall receptors, i.e. exerts the same fungicidal activity [12]. In particular, KP is the first engineered peptide able to maintain the microbicidal activity of native Ab through interaction with specific receptors in target microorganisms [13]. Notably, KP proved also to act, by different mechanisms, *in vitro*, *ex vivo* and/or *in vivo* against taxonomically unrelated pathogens, such as viruses (HIV and Influenza), bacteria, protozoa (*Leishmania* and *Achantamoeba*) and algae, other than yeasts, including strains resistant to conventional drugs as described in a number of further studies and reviewed in [14].

As no data have been provided so far, the aim of this study was the evaluation of the *in vitro* effect of KP against *C*. *albicans* biofilm and the definition of the molecular mechanisms possibly involved.

## Materials and methods

### Candida albicans

A total of six *C*. *albicans* strains were employed in this study, the reference strain SC5314, two wild-type clinical isolates (DSY544 and DSY347) and two clinical isolates which had been knocked out for their resistance mechanisms to fluconazole (DSY775 was derived from DSY544; DSY289 was derived from DSY347); details on the resistance-conferring alleles and knock-out procedures for the isolates DSY775, DSY544, DSY289 and DSY347 (kindly furnished by Dominique Sanglard from the University Hospital of Lausanne, Lausanne, Switzerland) have been described elsewhere [15–18]. Antifungal susceptibility profile of each isolate has been confirmed using the Etest method (bioMérieux, Marcy-l’Étoile, France). All the isolates were grown in Sabouraud Dextrose Agar (SDA) plates and maintained by biweekly passages.

In selected experiments, *C*. *albicans* SC5314 transformed with CIp10::ACT1p-gLUC59 plasmid (BLI *C*. *albicans*) was used [19]. It was grown in Yeast Peptone Dextrose Agar (YPD) and maintained by biweekly passages.

Before each experiment, a loop of cells from SDA or YPD was inoculated in sterile phosphate-buffered saline (PBS, EuroClone, Wetherby, UK), suspended in RPMI 1640 (Gibco, Grand Island, NY, USA) at 10^6^ cells/ml. This medium was supplemented with 10% heat inactivated foetal bovine serum (Defined Hyclone, Logan, Utah, USA), 50 mg/ml gentamicin (EuroClone,) and 2 mM L-glutamine (EuroClone), hereafter referred to as cRPMI.

### Synthetic peptides

The engineered synthetic decapeptide known as killer peptide (KP, AKVTMTCSAS) was employed in this study. A scrambled peptide (SP, MSTAVSKCAT), containing the same aminoacids of KP in a different sequence, was also included as a negative control [13]. Both peptides were synthesized by NeoMPS (PolyPeptide Group, Strasbourg, France)

### Evaluation of KP fungicidal activity against planktonic *C*. *albicans*

KP fungicidal activity was evaluated by a previously described colony forming unit (CFU) assay [20]. Briefly, approximately 500 viable yeast cells were incubated for 6 h at 37°C in the absence (control) or presence of KP, at decreasing concentrations, in 100 μl of distilled water. Yeast suspensions were then seeded on plates of SDA and colonies were enumerated after 48–72 h of incubation at 30°C. Percent killing was calculated with reference to the number of colonies in controls. Each assay was performed in triplicate. The half maximal effective concentration (EC_50_) was calculated by nonlinear regression analysis using Graph Pad Prism 4.01 software, San Diego, CA, USA.

### KP-mediated effects on *C*. *albicans* biofilm

In order to assess the effects of KP on the ability of *C*. *albicans* to produce biofilm, a fungal suspension (10^6^ cells/ml) was transferred (100 μl/well) into flat-bottom 96-well plates (Corning Incorporated, NY, USA) and incubated at 37°C.

To investigate KP effects on early stages of biofilm formation, after 90 min of *C*. *albicans* adherence, non-adherent fungal cells were removed and samples were untreated (ctrl) or treated with 100 μl of KP (31, 62 or 124 μg/ml) or SP (124 μg/ml) for 6 h; then, fresh cRPMI (100 μl/well) was added and the plates were further incubated at 37°C with 5% CO_2_ until 48 h, according to the standard protocol for biofilm production [21].

To investigate KP effects on mature biofilm, fungal cells were suspended in cRPMI (10^6^ cells/ml), then 100 μl/well were transferred in flat-bottom 96-well plates and incubated at 37°C for 48 h to allow biofilm formation and maturation. Samples were then treated with KP (31, 62 or 124 μg/ml) or SP (124μg/ml) for further 6 h at 37°C with 5% CO_2_ prior to be assessed for biofilm content. Untreated samples served as further negative controls.

Crystal Violet (CV) and 2,3-bis(2-methoxy-4-nitro-5-sulfophenyl)-2H-tetrazolium-5-carboxanilide (XTT, Sigma, St Louis, MO, USA) reduction-based assays were used, in order to quantify total biofilm mass and cell viability, respectively, as detailed elsewhere [22, 23].

The optical density was measured by a spectrophotometer (Sunrise^™^, Tecan Magellan) at 540 nm for the CV assay and at 450 nm (with 620 nm used as reference wavelength) for XTT assay. The results were expressed as percentage of biofilm and percentage of metabolic activity respectively in treated samples *vs* untreated controls [24, 25]. Moreover, EC_50_ was calculated with reference to biofilm inhibition assessed by CV assay.

### Measurement of KP-mediated oxidative stress response and membrane permeability in *C*. *albicans*

Yeast suspension (10^6^ cells/ml, 1 ml/well) was transferred into flat-bottom 24-well plates; each well containing a tissue culture coverslip (Corning Incorporated, NY, USA). Plates were then incubated for 48 h at 37°C with 5% CO_2_. After incubation, each well was washed with 1500 μl of sterile distilled water; then 500 μl/well of KP or SP (both at 124 μg/ml) were added and plates were incubated for additional 3 h. Cellular reactive oxygen species (ROS) production and intracellular ROS production were measured by CellROX^®^ Oxidative Stress Reagents fluorogenic probes (Molecular probes^®^, Life Technologies^™^) and by MitoSOX Red (Molecular probes^®^, Life Technologies^™^) respectively, according to the manufacturers’ instructions.

The fluorescence intensity was determined using a Nikon Eclipse 90i imaging system equipped with Nomarski DIC optics (Nikon Instruments Inc., USA). Images were acquired with a DS-2Mv Nikon digital camera, and the resulting photographs were analysed by using the Nikon NISELEMENTS version D3.1 software.

In parallel groups, fungal membrane permeability was assessed: briefly, samples treated as described above were washed and exposed to propidium iodide (PI, 1 mg/ml) for 15 min at 37°C. Samples were washed and immediately examined by fluorescence microscopy.

### Analysis of *C*. *albicans* biofilm-related gene expression after KP treatment

A qRT-PCR was used to investigate KP-induced changes in transcription level of genes related to biofilm formation.

Briefly, *C*. *albicans* SC5314 cells (10^7^ cells/ml) were grown in flat-bottom 6-well plates (Becton Dickinson Labware Europe, Meylan Cedex, France) for 1.5 h or 24 h at 37°C in cRPMI. Next, wells were washed and cells were treated with KP (124 μg/ml) for 1.5 h. At the end of the incubation time, wells were washed and adherent fungal cells were removed by gentle scraping. Total RNA was extracted from scraped cells with the Nucleospin RNA kit (Macherey Nagel, Duren, Germany) according to manufacturer's instructions and stored at -80°C. Quality and quantity of the extracted RNA were determined spectrophotometrically. Total RNA (1 μg) was converted into cDNA with random primers in a 20 μl reaction volume, using the Reverse Transcription System kit (Promega), following manufacturer's instructions. The transcription levels of *C*. *albicans ALS3*, *HWP1*, *ECE1*, *RAS1*, *EFG1*, *CYR1*, *HST7*, *GSC1*, *ZAP1*, *ADH5*, *CSH1* and *TEC1* genes were detected by using primers described elsewhere [26]. qRT-PCR mixtures contained 6 μl cDNA, 10 μl of SsoAdvanced^™^ universal SYBR^®^ Green supermix (BioRad, Milan, Italy), 0.5 pMol/μl of each primer, and sterile MilliQ water to a final volume of 20 μl. qRT-PCR was performed in 96-well plates on CFX96 Touch Real-Time PCR Detection System (BioRad) (95°C for 60 s, followed by 40 cycles of 95°C for 5 s, 60°C for 30 s). Actin (*ACT1*) was used as internal control. The transcription level of the selected genes was calculated using the formula of 2^-ΔΔCt^. Two independent experiments were performed, each in triplicate.

### Analysis of KP effects on a medical device-associated *C*. *albicans* biofilm

KP capability to interfere with *C*. *albicans* biofilm on medical devices was assessed using a central venous catheter (CVC). CVC preparation and pre-coating were performed according to the Kucharíková S. *et al* protocol [27] with minor modifications. Briefly, catheter was cut in pieces of exactly 0.4 mm and placed into micro-centrifuge tubes with 1.5 ml of foetal bovine serum; after vigorous vortex, samples were incubated at 37°C overnight. After incubation, serum-coated catheter pieces were transferred into flat-bottom 96-well plates and 200 μl/well of BLI *C*. *albicans* suspension (5 x 10^4^ cells/ml) were added and incubated with the CVC pieces for 1.5 h at 37°C in 5% CO_2_. Then, *C*. *albicans*-infected CVC pieces were employed in two different protocols. Firstly, to test the effect of KP on biofilm formation, *C*. *albicans-*infected CVC pieces were washed twice with PBS, seated into cRPMI (untreated ctrl) or treated with 200 μl of KP or SP (both 124 μg/ml) for 6 h at 37°C; then fresh cRPMI was added and samples were further incubated until 48 h at 37°C, in order to allow biofilm production. Secondly, to investigate KP effect on mature biofilm, *C*. *albicans*-infected catheter pieces were suspended in cRPMI for 48h, then they were washed twice with PBS and untreated (ctrl) or treated with KP or SP (both 124 μg/ml) for further 6 h at 37°C with 5% CO_2_. At the end of the incubation, CVC pieces were washed twice with PBS, transferred into black 96-well microtiter plates and exposed to 200 μl/well of 2 μM coelenterazine (Synchem, OHM) in luciferase assay buffer. Luciferase activity was then measured using a Luminometer (Victor Multilabel Plate Reader, PerkinElmer) and expressed as relative luminescence units (RLU).

After luciferase determination, CVC pieces were transferred in tubes containing 1 ml of PBS and sonicated for 15 min at 35,000 Hz in a water bath sonicator, placed on ice and vigorously vortexed for 30 sec. Finally, 100 μl of each sample were diluted 1/100 and plated on SDA, then incubated for 24 h at 37°C to evaluate CFU number.

### Statistical analyses

Data are depicted as the mean ± standard error (SEM) from triplicate samples of three independent experiments. Statistical analysis was performed using ANOVA with Bonferroni’s post-hoc test. For qRT-PCR data were analysed using Repeated Measures ANOVA test followed by Dunnett's Multiple Comparison Test. Values of *p*<0.05 and *p*<0.001 were considered significant.

## Results

### KP fungicidal activity against planktonic *C*. *albicans*

Preliminary experiments were carried out on planktonic yeasts by a CFU assay, using the *C*. *albicans* reference strain SC5314, two clinical isolates (DSY544 and DSY347) susceptible to fluconazole and their corresponding fluconazole-resistant counterparts (DSY775 and DSY289). KP proved to exert a significant fungicidal activity against all the investigated strains, with EC_50_ values ranging from 0.31 to 0.67 μM (Table 1).

**Table 1:** KP activity against planktonic yeast cells.

*C*. *albicans* strains	EC_50_* (95% confidence intervals) [mol/liter] ×10^−6^
SC5314 (wt)	0.67 (0.63–0.72)
DSY544 (s)	0.38 (0.30–0.48)
DSY775 (r)	0.41 (0.38–0.42)
DSY347 (s)	0.31 (0.27–0.35)
DSY289 (r)	0.50 (0.44–0.57)

*EC_50_, half maximal effective concentration, calculated by nonlinear regression analysis using Graph Pad Prism 4.01 software

### Inhibitory effects of KP on early stages of *C*. *albicans* biofilm development

Firstly, we investigated the capability of KP to interfere with *C*. *albicans* biofilm formation onto polystyrene plates. All the described *C*. *albicans* strains were used. *C*. *albicans* yeast cells, pre-incubated in 96 well plates (10^5^/well, in cRPMI) at 37°C for 90 min, were untreated or treated for 6 h to increasing doses of KP (31, 62 or 124 μg/ml) or with SP (124 μg/ml) as a negative control. Fresh cRPMI was then added and samples were further incubated up to 48 h. Biofilm mass and metabolic activities were then evaluated by CV and XTT assays, respectively.

**Fig 1:**
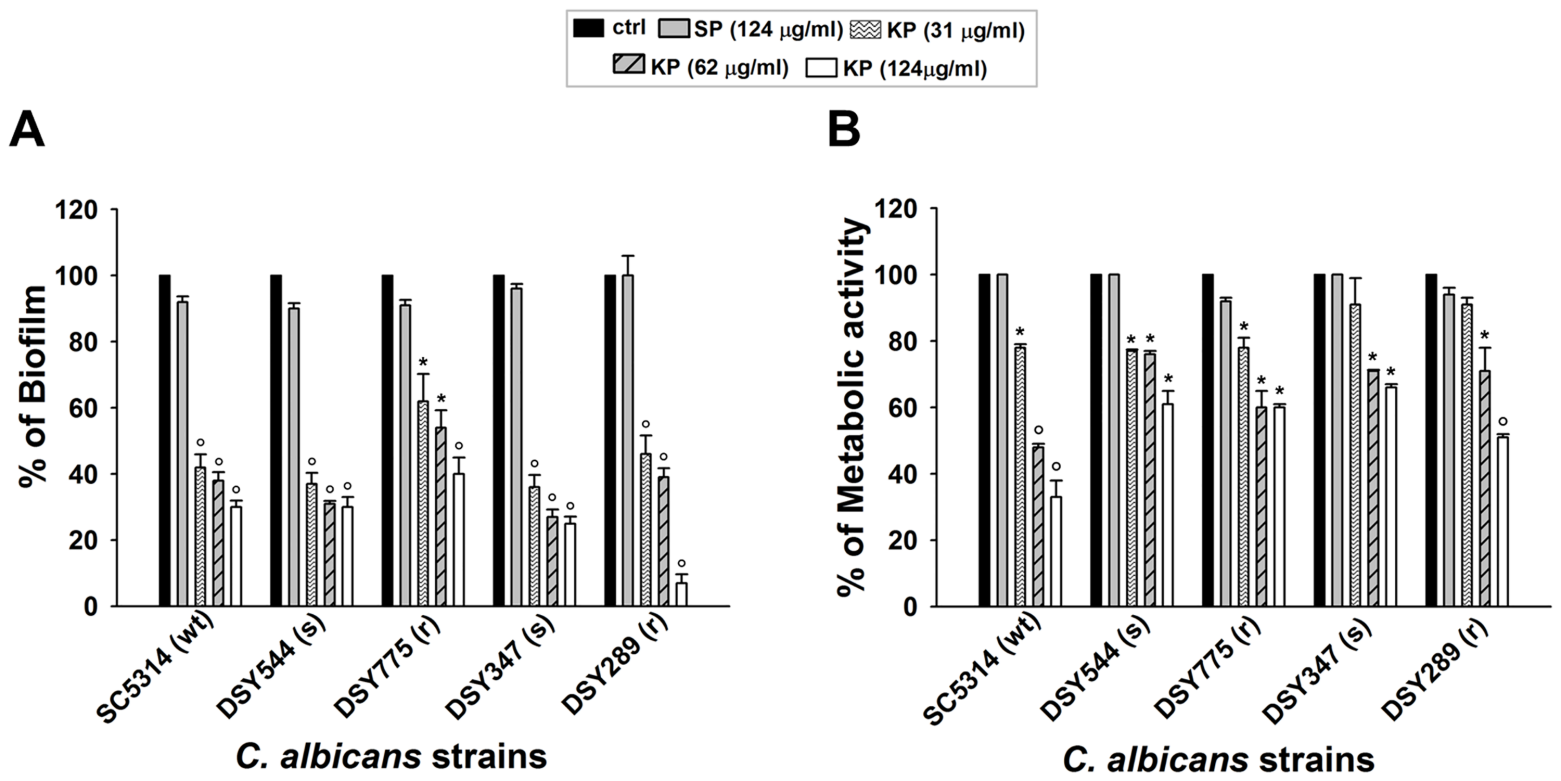
Effect of KP on early stages of *C*. *albicans* biofilm development. Yeast cells were suspended in cRPMI and seeded in 96-well plates (10^5^/well). After 90 min of incubation, samples were washed and untreated (ctrl) or treated for 6 h with increasing doses of KP (31, 62 or 124 μg/ml) or with SP (124 μg/ml); then, fresh cRPMI was added and plates were incubated up to 48 h to allow biofilm formation. Crystal violet staining (A) and XTT assay (B) were performed. Data are expressed as percentage of biofilm (A) and percentage of metabolic activity (B) of treated samples compared to untreated controls (100%). *p<0.05 or °*p*<0.001 indicate significant differences between KP-treated *vs* untreated groups.

KP was found to significantly reduce both biofilm mass, expressed as percentage of biofilm formation (Fig 1A) and metabolic activity (Fig 1B); the effects were dose-dependent and similar trends were observed among each of the five *C*. *albicans* strains tested. In particular, it should be noted that the KP dose of 124 μg/ml consistently impaired biofilm, reducing total biomass by more than 45% in four strains and down to 10% in DSY289 (Fig 1A). EC_50_ values, calculated with reference to biofilm inhibition assessed by CV assay, are shown in Table 2. Concerning the metabolic activity (Fig 1B), all the strains tested showed a significant reduction, ranging between 33% (SC5314) and 66% (DSY347) when exposed to 124 μg/ml of KP. Notably, no inhibitory effects were ever observed upon SP treatment; both the percentage of biofilm formation and the percentage of metabolic activity were indeed very close to those of control groups, remaining close to 100%.

**Table 2:** KP activity against *C*. *albicans* biofilm.

	EC_50_* [mol/liter] ×10^−6^	
*C*. *albicans* strains	biofilm formation	mature biofilm
SC5314 (wt)	14.07	137.55
DSY544 (s)	2.75	182.83
DSY775 (r)	70.73	694.47
DSY347 (s)	6.49	136.25
DSY289 (r)	31.03	159.59

*EC_50_, half maximal effective concentration, calculated by nonlinear regression analysis using Graph Pad Prism 4.01 software

### Inhibitory effects of KP on mature *C*. *albicans* biofilm

**Fig 2:**
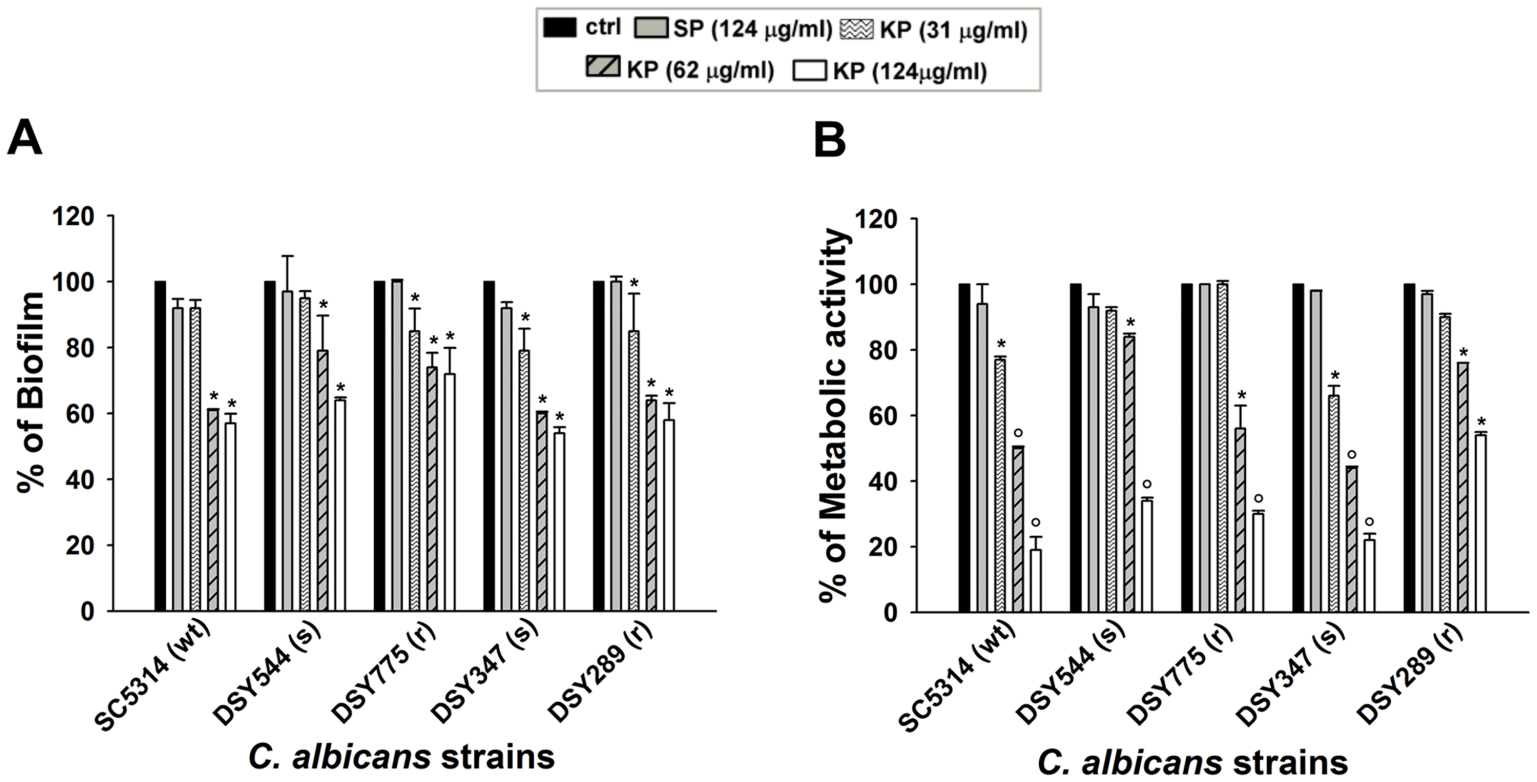
Effect of KP on mature *C*. *albicans* biofilm. Forty-eight h-old biofilms obtained from 5 different *C*. *albicans* strains were exposed to cRPMI (untreated) or treated with increasing doses of KP (31, 62 or 124 μg/ml) or with SP (124 μg/ml) for 6 h. Then, total biofilm biomass was measured by crystal violet staining (A) and metabolic activity was assessed by XTT assay (B). Data are expressed as percentage of biofilm (A) and percentage of metabolic activity (B) in treated samples compared to untreated controls (100%). *p<0.05 or °*p*<0.001 indicates significant differences between KP-treated *vs* untreated groups.

The capacity of KP to affect mature biofilm was also evaluated. Mature biofilm (48 h), obtained with each of the five *C*. *albicans* strains employed, was either untreated (ctrl) or treated with KP or SP, as described above. Hence, biofilm was analysed by CV and XTT assays. Our results showed that mature biofilms were susceptible to KP, but not to SP, irrespective of the strains employed and of their resistance or susceptibility to fluconazole. In particular, the highest dose of KP (124 μg/ml) significantly impaired both total mature biofilm biomass (Fig 2A) and metabolic activity (Fig 2B) as compared to those observed in untreated ctrls. Conversely, no inhibitory effects were ever observed in SP-treated biofilm. EC_50_ values, calculated with reference to biofilm inhibition assessed by CV assay, are shown in Table 2.

### Oxidative stress response in mature *C*. *albicans* biofilm exposed to KP

**Fig 3:**
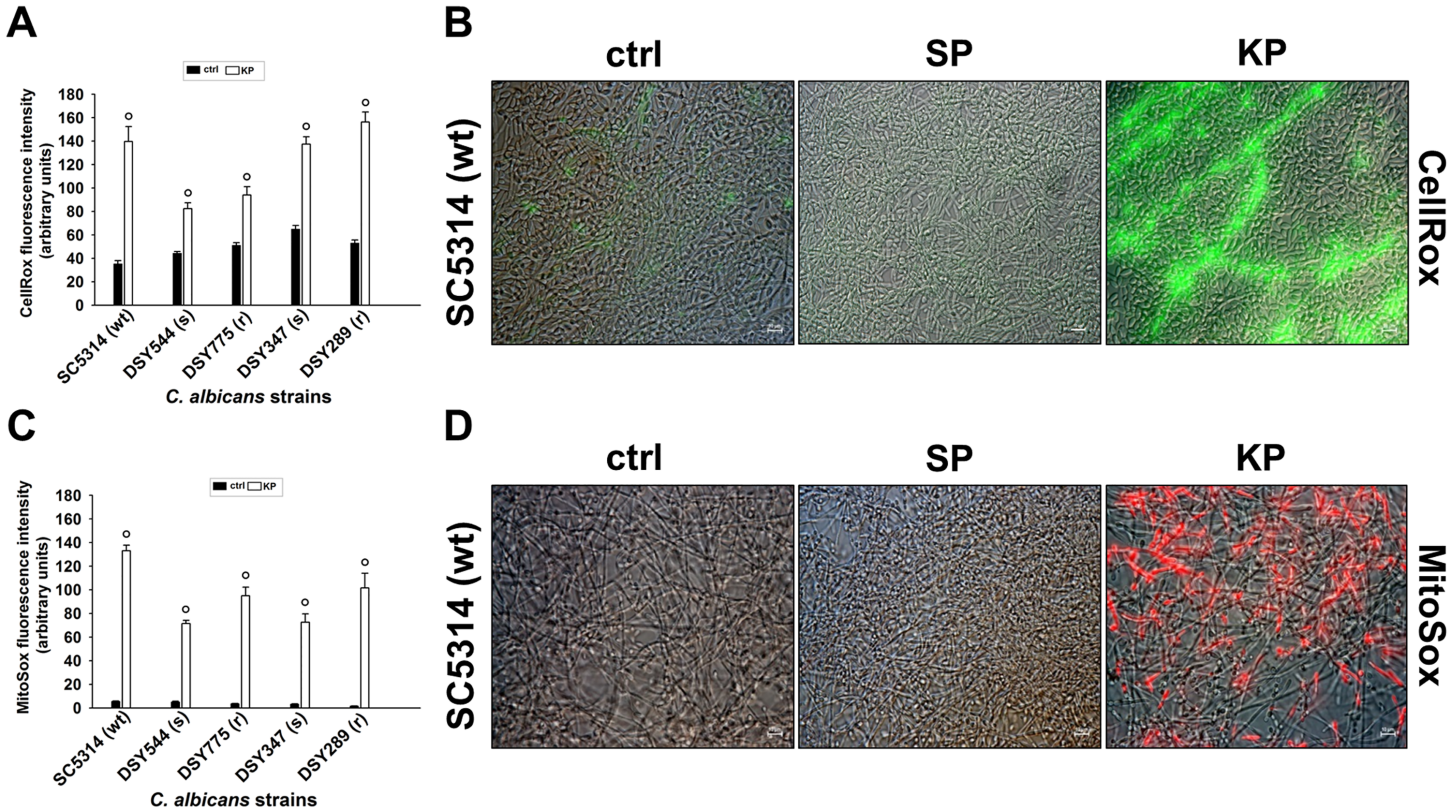
Reactive oxygen species production by *C*. *albicans* mature biofilm exposed to KP. Mature biofilms (48 h) by each of the 5 *C*. *albicans* strains employed were incubated in cRPMI (untreated ctrl) or treated with KP or SP (both at 124 μg/ml) for 3 h, then CellRox (A) and MitoSox (C) production were analysed by fluorescent microscopy. Data are expressed as arbitrary units of CellRox (green) or MitoSox (red) fluorescence intensity. Microphotographs from a representative experiment where CellRox (B) or MitoSox (D) production had been assessed are presented; mature biofilm (by the reference *C*. *albicans* strain SC5314) was untreated (ctrl) or treated with KP or SP (both at 124 μg/ml) and then investigated for ROS production. °*p*<0.001 indicates significant differences between KP-treated *vs* untreated groups.

Based on the above results, we selected the highest dose of KP (124 μg/ml) for the subsequent experiments. In order to investigate KP mechanisms of action against *C*. *albicans* biofilm, we tested the effects of KP on mature biofilm in terms of ROS and O_2_^-^ production. Briefly, 48 h-old mature biofilms obtained with each of the 5 *C*. *albicans* strains employed were untreated (ctrl) or treated with KP or SP (both 124 μg/ml) for 3 h; then, ROS production was measured by means of CellRox and MitoSox. Fig 3A shows that each strain spontaneously produced detectable levels of ROS; moreover, KP treatment significantly increased ROS production and, although to a different extent, the phenomenon occurred with each of the strains tested. No effect was observed after SP treatment (data not shown). Fig 3B shows a representative image of CellRox production in a 48-h biofilm (by the reference *C*. *albicans* strain SC5314) that had been untreated (ctrl) or treated with KP or SP (both 124 μg/ml). As detailed in the figure, control groups (untreated ctrl and SP-treated biofilm) consisted of a densely packed biofilm exhibiting poorly detectable fluorescence. Differently, KP treatment resulted in a potent induction of fluorescence: in particular, the green signal was very intense and mostly aggregated along longitudinal lines, possibly consisting of adjacent yeasts and hyphae.

As shown in Fig 3C, O_2_^-^ production, almost undetectable in control groups, increased up to 70-fold upon KP treatment, while, once again, no effects were observed after SP treatment (data not shown). Fig 3D shows a representative image of MitoSox production by a 48-h biofilm of the reference *C*. *albicans* strain SC5314, untreated (ctrl) or treated with KP or SP (both 124 μg/ml). MitoSox staining revealed undetectable fluorescence in ctrl and SP-treated biofilm, while, in KP-treated biofilm, a diffused red fluorescent signal was evident, quite homogeneously distributed and possibly involving most of the cells.

### Cell viability reduction in mature *C*. *albicans* biofilm exposed to KP

**Fig 4:**
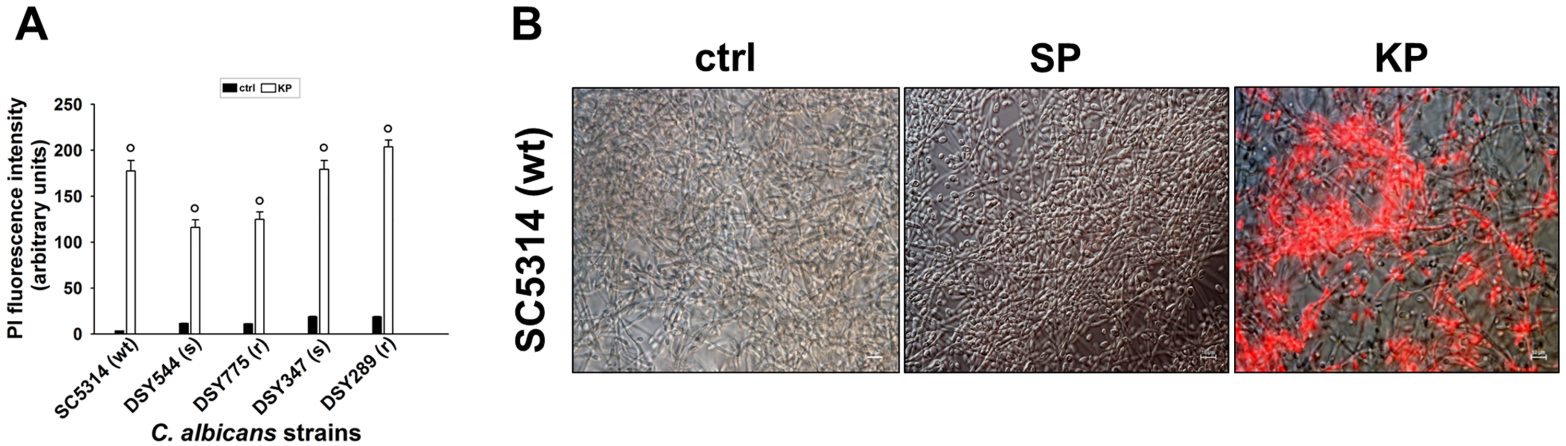
Cell viability following KP treatment of *C*. *albicans* mature biofilm. Mature biofilms (48 h) from each of the 5 *C*. *albicans* strains employed were untreated (ctrl) or treated with KP or SP (both 124 μg/ml) for 3 h, then PI staining was analysed by fluorescent microscopy. Data are expressed as arbitrary units of PI (red) fluorescence intensity (A). Microphotographs from a representative experiment of PI staining (B) in *C*. *albicans* mature biofilm (by the reference *C*. *albicans* strain SC5314) untreated (ctrl) or treated with KP or SP (both 124 μg/ml) are shown. °*p*<0.001 indicates significant differences between KP-treated *vs* untreated groups.

Next, we investigated the effects of KP treatment on fungal cell membrane permeability through the assessment of propidium iodide (PI) staining, a parameter used to measure cell viability. Our results (Fig 4A) showed that KP treatment significantly increased PI fluorescence intensity of *C*. *albicans* biofilm, as compared to the untreated counterparts and that such phenomenon occurred to a similar extent, irrespectively of the strain employed. Conversely, SP-treatment did not modulate PI staining (data not shown). Fig 4B shows representative microphotographs of a 48-h biofilm (by the reference *C*. *albicans* SC5314) either untreated (ctrl) or treated with KP or SP (both at 124 μg/ml). As detailed in the figure, PI fluorescent intensity (Fig 4A) appeared almost everywhere in biofilm treated with KP, with highly positive areas, recalling what was observed with the CellRox staining. No PI fluorescence was observed in untreated (ctrl) or SP-treated biofilm.

### KP affects transcriptional profiles of biofilm-associated *C*. *albicans* genes

**Fig 5:**
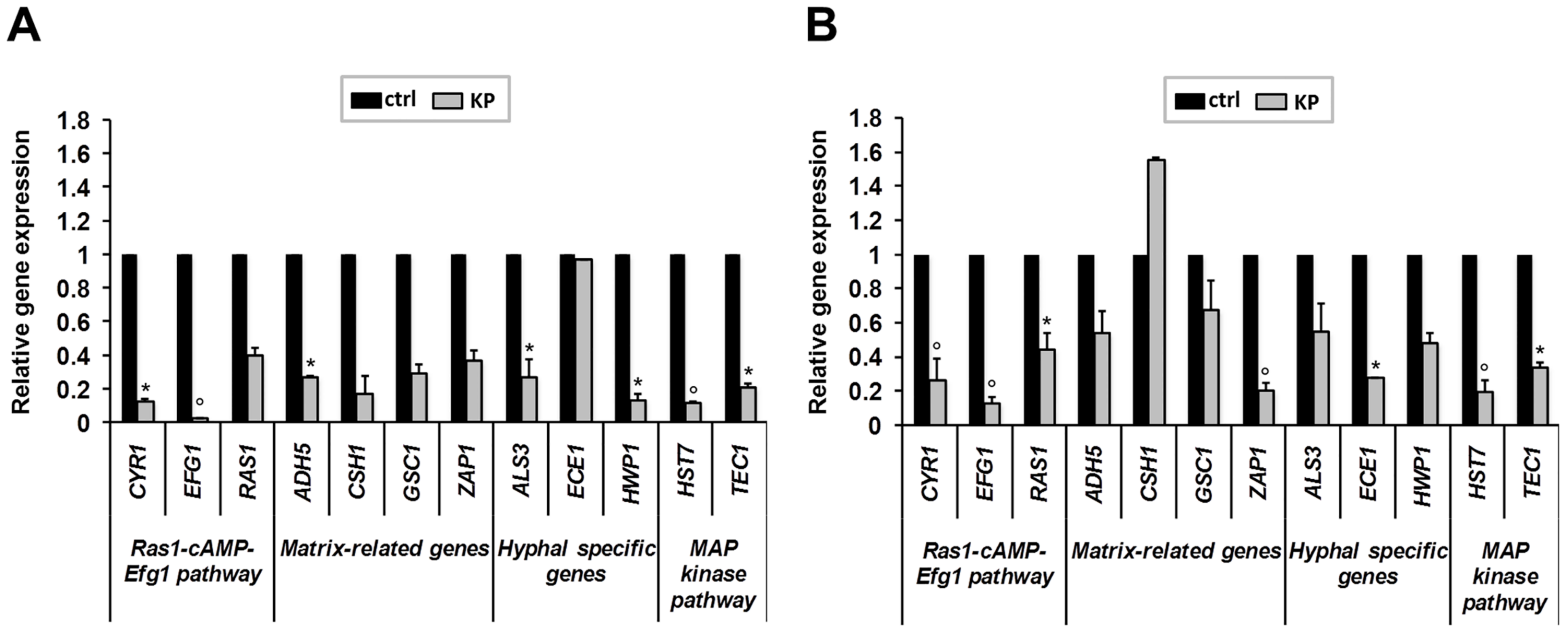
Quantitative RT-PCR analysis of *C*. *albicans* biofilm-related genes after KP treatment. *C*. *albicans* biofilms, at early (90 min in cRPMI; panel A) or late (24 h in cRPMI; panel B) stages of development, were exposed or not to KP (124 μg/ml for additional 90 min) and then assessed by qPCR analysis. The relative levels of gene expression are presented as fold changes in KP-treated groups (grey columns) with respect to the untreated controls (black columns). All gene transcript levels were normalized against *ACT1* gene expression. Statistically significant results were evaluated by Repeated Measures ANOVA test, followed by Dunnett's Multiple Comparison Test. *p<0.05 or *p*<0.001 indicates significant differences between KP-treated and untreated groups.

KP effects on the transcriptional profiles of 12 biofilm-associated genes were evaluated by quantitative RT-PCR using the *C*. *albicans* reference strain SC5314. KP treatment (124 μg/ml for 90 min) was performed either at early stages of biofilm formation (*C*. *albicans* was incubated with cRPMI for 90 min) or on 24 h-old biofilm. Then, samples were processed for molecular analysis. When used during early stages of biofilm development, KP treatment significantly reduced *CYR1*, *EFG1*, *ADH5*, *CSH1*, *ALS3*, *HWP1*, *HST7* and *TEC1* gene expression (Fig 5A); moreover, when used against mature biofilm, KP treatment significantly down-regulated *CYR1*, *EFG1*, *RAS1*, *ZAP1*, *ECE1*, *HST7* and *TEC1* expression (Fig 5B).

### KP impaired *C*. *albicans* biofilm formation and mature biofilm produced onto CVC

**Fig 6:**
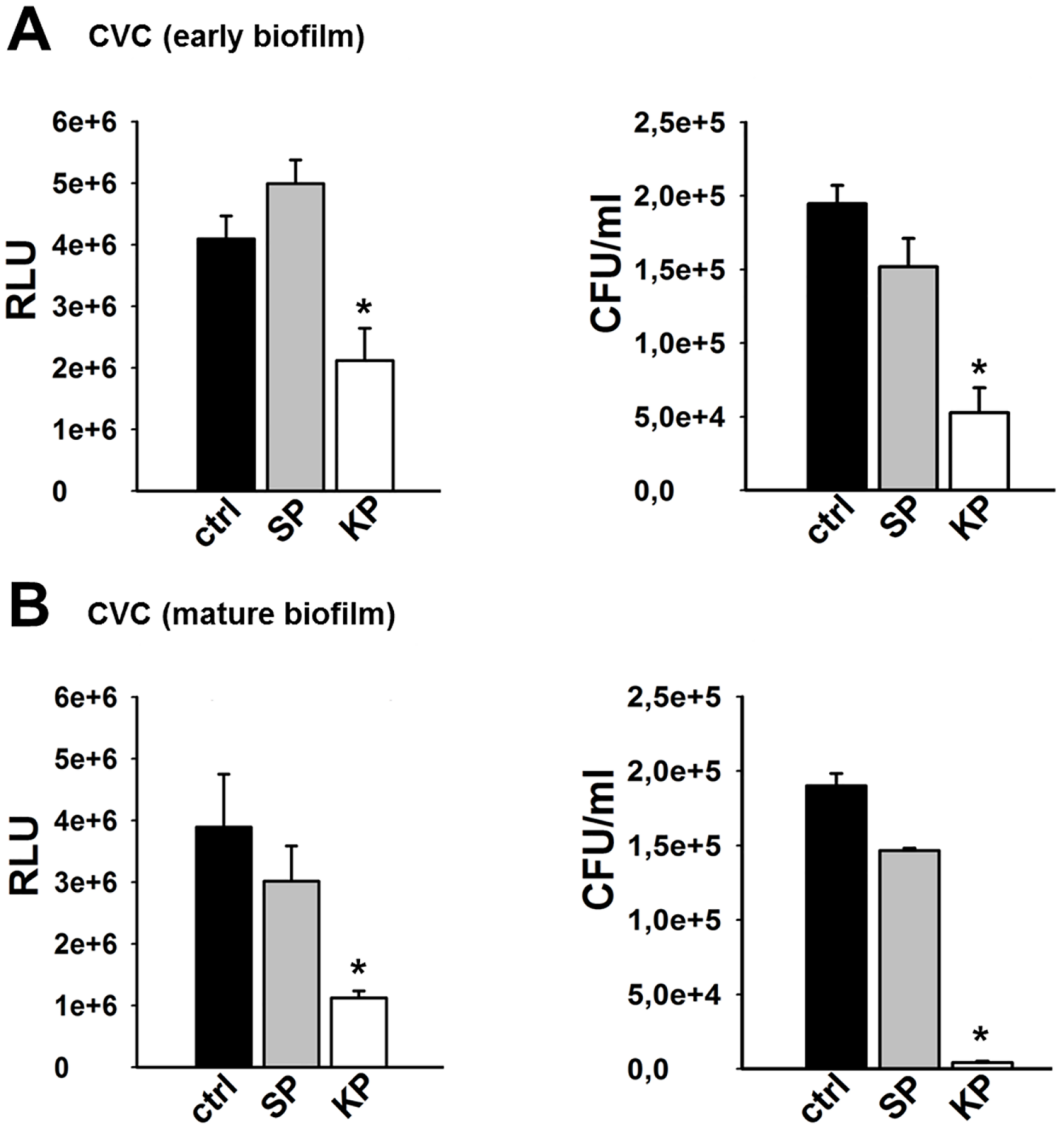
Evaluation of KP activity against *C*. *albicans* biofilm produced on CVC. Serum-precoated CVC pieces were exposed to BLI *C*. *albicans* (10^4^ cells/well of cRPMI, for 90 min at 37°C); after washing, they were untreated (ctrl) or treated for 6 h with KP or SP (both 124 μg/ml) and further incubated up to 48 h to allow biofilm formation (A). In further experiments, serum-precoated CVC pieces were exposed to BLI *C*. *albicans* (10^4^ cells/well of cRPMI) for 48 h at 37°C, prior to be untreated (ctrl) or treated with KP or SP (both 124 μg/ml) for further 6 h (B). In both cases, bioluminescence by residual biofilm was measured and expressed as RLU (left panels) as well as viable *Candida* cells were quantified by counting the CFU/ml (right panels). *p<0.05 indicates significant differences between KP-treated and untreated groups.

Since KP was shown to strongly reduce *C*. *albicans* biofilm formation and also to significantly impair mature biofilm, we wondered whether KP could also affect a medical device-associated fungal biofilm. Accordingly, a well-established model of *C*. *albicans* biofilm development on CVC was used, employing a bioluminescent (BLI) *C*. *albicans* strain [27]. As shown in Fig 6A, KP treatment significantly inhibited biofilm formation on CVC, as demonstrated by i) the decrease in relative luminescent units (RLU) emitted by live *Candida* cells and ii) the reduction of CFU number. Interestingly, KP was also able to significantly impair a mature biofilm, as detailed in Fig 6B. Also in this case, both the bioluminescent data and the CFU counts provided congruent results. In contrast, no effects were ever observed when using SP as negative control, irrespective of the assay used.

## Discussion

The human commensal yeast *C*. *albicans* can behave as an opportunistic pathogen in susceptible individuals, where it is isolated as the most common species responsible of superficial as well as invasive fungal infections. One of its most intriguing and poorly understood pathogenic peculiarities is its capability to organize in a biofilm on abiotic surfaces, such as implanted medical devices (i.e. prostheses, heart valves and catheters) as well as on mucosal surfaces, including oral and vaginal epithelia [28]. Once embedded in a biofilm, *C*. *albicans* cells become refractory to antifungal agents and host mediated defences, often causing failure of conventional therapy [29]. It follows an urgent need for new antimicrobial tools, effective against biofilm-associated organisms.

It has extensively been demonstrated that KP exerts anti antibacterial, antifungal, antiprotozoan, antiviral and immunomodulatory activity *in vitro*, *ex vivo* and/or *in vivo* by different mechanisms of action [14]. In particular, KP is effective against several fungal pathogens, including *Candida* spp., irrespective of their profile of resistance to conventional antifungal agents. Fungicidal activity involves interaction between KP and superficial molecules, such as β-1-3-ᴅ-glucan, while the down-stream occurring molecular events/mechanisms have not yet been clarified in depth [14]. Here, we provide the first evidence that KP is able to interfere with *C*. *albicans* biofilm. As established by CV and XTT assays, both total biomass and residual metabolic activity are significantly affected upon fungal exposure to KP. We demonstrate that not only KP is effective in preventing biofilm formation but also that it significantly impairs an already structured *C*. *albicans* biofilm. Notably, similar trends of inhibitory effects have been observed using the reference laboratory strain as well as four clinical isolates. As predictable, EC_50_ values calculated with reference to biofilm inhibition assessed by CV assay were much higher than the values of EC_50_ obtained on planktonic yeasts. Nevertheless, the present findings expand the knowledge on antifungal potential of KP, showing that it is capable of affecting also *C*. *albicans* biofilm, either at early stages formation or lately when a sessile community is already structured. In line with these data, initial evidence exists on the ability of other antimicrobial peptides to impair *C*. *albicans* biofilms [26, 30, 31]. In particular, human lactoferrin-11 synthetic peptide (hLF1-11) significantly inhibits biofilm formation, interfering with fungal cell density and metabolic activity; also *C*. *albicans* morphogenesis is affected through Ras1-cAMP-Efg1 pathway [26]. Taken together, these data strongly support the hypothesis of a peptide-based therapy as a novel strategy against biofilm-associated *C*. *albicans* infections either alone or in combination with conventional antimycotics and/or antimicrobial agents.

It is well known that under adverse conditions, such as in the presence of oxidants or upon exposure to antifungal drugs [32], yeast cells react producing free radicals, that in turn exacerbate cell damage, altering proteins, lipids, and DNA, eventually leading to cell death [33]. Noteworthy, also plant defensins have been found to inhibit fungal biofilms through similar mechanisms [34, 35]. Our results show that KP induces oxidative stress response and membrane damage within *C*. *albicans* biofilm. In particular, ROS and specifically mitochondrial superoxide as well as PI cell fluorescence significantly increase in KP-treated biofilm as compared to KP-untreated biofilms. The phenomenon occurs irrespective of the timing of biofilm exposure to KP (in early as well as in mature biofilm) and independently upon genetic drug-resistance/susceptibility of the *C*. *albicans* isolates employed. Thus, the present findings provide initial insights indicating that ROS production occurs and is likely involved in KP-induced *C*. *albicans* biofilm damage. In recent studies, the formation of ROS by yeast cells has been described as one of the crucial steps involved in the fungicidal mechanism of a number of peptides including histatin 5, lactoferrin-(1–11) and cathelicidin LL-37 [10, 36, 37]. Taken together, the previous and present data strengthen the wide-range potential impact of KP as a novel anti-fungal tool, irrespective of the planktonic or sessile organization of the pathogen.

Besides the ATCC reference strain, our model includes four *C*. *albicans* clinical isolates, known to differ in terms of susceptibility to fluconazole [15–18]. The fact that KP comparably affects the two susceptible (DSY544 and DSY347) and the two resistant (DSY775 and DSY289) isolates implies that its mechanisms of action are independent upon the fluconazole-resistance pathway. Although far from being elucidated, this phenomenon underlines the potential relevance of KP as a universally active anti-biofilm tool, likely not affected by either conventional or novel drug-resistance phenotypes.

Increasing literature details the transcriptional profiles of genes associated with the distinct stages of *C*. *albicans* biofilm formation, such as cell adhesion, hyphal formation and extracellular matrix production [28, 38, 39]. Here, we show that KP affects biofilm gene expression, when added during the early stage of biofilm formation as well as when used onto mature biofilm. In both conditions, the expression levels of Ras1-cAMP-Efg1 pathway appear significantly reduced. It should be noted that Efg1 transcriptional factor is activated by *RAS1* and *CYR1* and also that it controls some hyphae-specific genes [40]; therefore, any alteration occurring at the level of Efg1 implies a complex and likely wide series of down-stream events. The inhibitory effects observed in our *C*. *albicans* biofilm model closely recall recent observations showing that other antimicrobial peptides, such as hLF1-11 [26] and tetrandrine [41], result in a significant decrease in the expression levels of this same gene pathway. In our hands, also *HST7* and *TEC1* genes, known to be involved in the MAP kinase pathway and in the expression of hyphae-specific genes [42, 43], are significantly down-regulated after KP treatment, irrespective of the biofilm formation stage. Furthermore, also the hyphae-specific genes tested (*ALS3*, *ECE1*, *HWP1*), encoding for molecules essential to *C*. *albicans* adhesion and biofilm formation [28, 38, 39], are down-regulated after KP treatment both in early and mature biofilm.

Concerning extracellular matrix production, all the genes tested are down-regulated in early stages of biofilm formation, while a different pattern of expression is observed in KP-treated mature biofilm. According to the literature [44, 45], the main extracellular carbohydrate in *C*. *albicans* is β-1,3-ᴅ-glucan and one of the genes involved in its synthesis is *GSC1*. During early stages of biofilm formation, exposure to KP significantly down-regulates *GSC1* transcript, while the observed KP-induced negative regulation is reduced in mature biofilm. A similar trend is found for *ADH5*, while *CSH1* transcriptional levels increase upon KP treatment even though not attaining statistical significance. Since extracellular matrix is most abundantly produced at the beginning of biofilm formation [28], we may expect that matrix-associated genes, being more actively expressed in a young biofilm are indeed more susceptible to KP down-regulation during early matrix formation rather that at later stages in a mature biofilm. The only exception to this happens to be the *ZAP1* gene, that indeed is profoundly down-regulated at both time points.

Finally, the antifungal effects of KP have been demonstrated against a *C*. *albicans* biofilm developed onto CVC *in vitro*. To our knowledge, this is the first evidence on the efficacy of KP also against medical device-associated microorganisms already structured in a biofilm.

In conclusion, our results provide the first evidence on the anti-biofilm activity of KP and on the possible events/mechanisms involved in such phenomenon. The comparable inhibitory effects of KP against drug-susceptible and drug-resistant isolates opens to the idea of proposing such a tool as novel antifungal strategy, likely effective independently upon the pathogen’s drug-resistance profile. Furthermore, the retained efficacy of KP against CVC-associated biofilm offers the rational for future applications of KP directly onto surfaces of medical devices in the attempt to contain the risk of device-associated invasive candidiasis. The therapeutic potential of cationic antimicrobial peptides (AMPs) is already being explored, demonstrating the efficacy of synthetic peptides in phase III clinical trials for prevention of catheter-associated infections [46]. So far, the most investigated AMPs categories for clinical purposes are lantibiotics, temporins, cathelicidins and defensins. As a matter of fact, the knowledge that fragments derived from both the variable and constant regions of immunoglobulins may exert a fungicidal activity similar to the one displayed by KP [20, 47, 48], regardless of their specificity and isotype, further extend the potential significance of antibody-derived peptides.
